# The Global Trends and Advances in Oral Microbiome Research on Oral Squamous Cell Carcinoma: A Systematic Review

**DOI:** 10.3390/microorganisms13020373

**Published:** 2025-02-08

**Authors:** Ramona Dumitrescu, Vanessa Bolchis, Aurora Doris Fratila, Daniela Jumanca, Berivan Laura Rebeca Buzatu, Ruxandra Sava-Rosianu, Vlad Tiberiu Alexa, Atena Galuscan, Octavia Balean

**Affiliations:** 1 Translational and Experimental Clinical Research Centre in Oral Health, University of Medicine and Pharmacy “Victor Babes”, 300040 Timisoara, Romania; dumitrescu.ramona@umft.ro (R.D.); jumanca.daniela@umft.ro (D.J.); berivan.haj-abdo@umft.ro (B.L.R.B.); sava-rosianu.ruxandra@umft.ro (R.S.-R.); vlad.alexa@umft.ro (V.T.A.); galuscan.atena@umft.ro (A.G.); balean.octavia@umft.ro (O.B.); 2 Clinic of Preventive, Community Dentistry and Oral Health, University of Medicine and Pharmacy “Victor Babes”, Eftimie Murgu Sq. no 2, 300041 Timisoara, Romania; 3 Faculty of Dental Medicine, Ludwig Maximilian University of Munich, Goethestrasse 70, 80336 Munich, Germany; a.fratila@campus.lmu.de

**Keywords:** VOSviewer, microbiome, OSCC, salivary biomarkers

## Abstract

The oral microbiome is increasingly recognized as a key factor in the development and progression of oral squamous cell carcinoma (OSCC). Dysbiosis has been associated with inflammation and tumorigenesis, highlighting the potential of microbial alterations and salivary biomarkers as tools for early, non-invasive diagnosis. This review examines recent advancements in understanding the oral microbiome’s role in OSCC. A comprehensive synthesis of studies from 2016 to 2024 was conducted to identify emerging themes and significant findings in the field. Key topics included the interplay between microbiome-driven mechanisms and cancer development, with a focus on microbial communities and their metabolic byproducts. The findings emphasize the importance of specific microbial alterations in modulating immune responses and tumor microenvironments, as well as the promise of biomarkers such as interleukins and miRNA signatures in improving diagnostic accuracy. Recent research trends indicate growing interest in the therapeutic potential of targeting the oral microbiome in OSCC management. Despite significant advancements, gaps remain in the understanding of the precise mechanisms linking dysbiosis to cancer progression. This review underscores the need for continued research to develop personalized diagnostic and therapeutic strategies based on the oral microbiome, with the potential to transform OSCC management.

## 1. Introduction

The term “microbiome” was coined by Joshua Lederberg, a Nobel Prize laureate, to denote the diverse community of symbiotic, commensal, and pathogenic microorganisms residing within our bodies [1]. These microorganisms collectively inhabit our body space, contributing to an intricate ecological system. Remarkably, the abundance of microbes in our bodies rivals, if not exceeds, that of our own cells [2]. Among these microbiomes, the oral microbiome specifically refers to the array of microorganisms inhabiting the oral cavity of humans.

First recognized in 1674 by Antony van Leeuwenhoek, the oral microbiome has since become a key focus in microbial ecology, with research significantly advancing to reveal its crucial impact on both local and systemic health [3,4].

Recent advancements in salivary metabolomics have highlighted the potential of salivary biomarkers for the early detection and monitoring of various diseases. Salivary metabolites, derived from both oral microbes and host sources, provide valuable insights into physiological and pathological processes [5]. Saliva is a valuable biological fluid containing a diverse array of biomarkers derived from serum, gingival crevicular fluid, and oral microorganisms and their products, making it an attractive option for laboratory tests due to its non-invasive collection, ease of transportation and storage, cost-effectiveness, and efficiency [6,7,8]. Various molecular techniques are employed to detect and determine biomarkers, including DNA microarrays, the polymerase chain reaction (PCR), liquid chromatography, mass spectrometry, and nuclear magnetic resonance, among others. Oral squamous cell carcinoma (OSCC), a prevalent form of oral cancer, is characterized by significant diagnostic challenges due to its asymptomatic nature in early stages and poor 5-year survival rates, which remain unimproved despite advanced combination treatments such as surgery, chemotherapy, and radiotherapy. OSCC development has been closely linked to the oral microbiome, which triggers inflammatory responses through cytokines and chemokines, stimulating tumor cell proliferation and survival, emphasizing the importance of identifying salivary biomarkers for early diagnosis and improved prognosis [9].

Traditional diagnostic methods often fail to detect OSCC until it reaches advanced stages, underscoring the need for non-invasive and reliable biomarkers. Our study investigates the salivary metabolome of OSCC patients, identifying key metabolites that distinguish cancerous from non-cancerous states and exploring their potential as diagnostic and prognostic indicators. Salivary biomarkers have gained significance in the screening and early detection of oral squamous cell carcinoma (OSCC), with over 100 potential biomarkers reported in the literature. These biomarkers can be classified based on disease state, biomolecules, or other criteria, with diagnostic markers such as EFNB2 gene expression, interleukins (IL-6 and IL-8), and 8-oxoguanine DNA glycosylase being of particular interest for OSCC screening and prognosis evaluation. For IL-6, Rani et al. (2023) [10] reported a sensitivity of 85% and specificity of 78% (AUC = 0.82), demonstrating its diagnostic utility for OSCC. Similarly, salivary transferrin has shown an AUC of 0.89 in early-stage OSCC detection (Tavakoli et al., 2024 [11]). These findings highlight the potential of these biomarkers for non-invasive diagnostic applications. Recent efforts have focused on non-invasive methods for understanding OSCC genomic architecture, utilizing proteomic, transcriptomic, and metabolomic biomarkers extracted from human saliva samples [12].

Despite ongoing efforts in early screening and diagnosis, the burden of oral cancer treatment is expected to increase significantly due to factors such as its asymptomatic nature in early stages, which often leads to late diagnosis, and the limited improvements in survival rates despite advances in surgery, chemotherapy, and radiotherapy [13,14].

According to the latest data from the Institute for Health Metrics and Evaluation (IHME) on the global burden of disease (GBD), oral cancer poses a significant global health concern, with an estimated 373,000 incident cases, 199,000 deaths, and 5.51 million disability-adjusted life years (DALYs) in 2019 alone [15]. Research efforts towards oral cancer have been continuously rising, covering various aspects including diagnostic, prognostic, and therapeutic modalities, as well as qualitative and health system analyses.

The complex etiology of cancer involves both environmental and heritable risk factors, with increasing evidence suggesting that the microbiome plays a key role in modulating the carcinogenic process through distinct mechanisms. These include microbiome-driven dysbiosis leading to immune evasion, the production of pro-inflammatory cytokines such as IL-6 and TNF-α that contribute to chronic inflammation, and microbial metabolites influencing oncogenic pathways. While the microbiota harbors beneficial effects including active communication with host cells and maintenance of an environment rich in nutrients, it also poses risks by producing toxins that can induce mutations, alter signaling pathways, and promote cancer development in both oral and systemic contexts [16].

Given the accessibility of the oral cavity for non-invasive biological sampling, salivary-based diagnostic tools are increasingly being explored for their potential in early OSCC detection and personalized risk assessment. Future research should focus on refining these biomarkers to improve sensitivity and specificity, ultimately contributing to precision medicine approaches in oncology [17].

Bibliometric analysis [18] objectively evaluates extensive data from the past to quantify a study’s impact within its specific scientific field. Bibliometric analysis plays a crucial role in assessing, evaluating, and visualizing research trends and evidence within a specific field, thereby enabling future researchers and stakeholders to make informed decisions regarding research priorities [19,20].

A thorough bibliometric analysis should encompass both performance analysis, which quantifies citation and impact counts, and science mapping, which examines the key trends, topics, and collaboration networks among authors, affiliations, and countries involved [21].

Although many studies have explored the link between bacteria and oral cancer, no bibliometric analysis has been conducted on this topic. The oral microbiome’s impact on systemic health is gaining attention, as microbial metabolites influence both local and systemic conditions. Salivary metabolic profiling provides insights into disease mechanisms and potential therapies. By examining the salivary metabolome’s role in OSCC and its systemic implications, our research enhances the understanding of salivary metabolomics and its clinical applications. In this study, we conducted a comprehensive bibliometric analysis focusing on the literature published in the last eight years. Our analysis encompassed article citations, countries of origin, publishing journals, authors and their affiliations, highly cited studies, and keywords. Additionally, we explored research trends and identified hotspots. The aim of this analysis is to develop a set of salivary biomarkers for the oral microbiome and cytokines, with the aim of evaluating OSCC patients based on variations in these parameters, in the meantime offering researchers an unbiased comprehension of the research landscape in this domain, and to serve as a guide for future in-depth inquiries [12,22,23,24].

## 2. Materials and Methods

### 2.1. Data Sources, Collection, and Processing

The methodology employed in the present study’s analysis is derived from the bibliometric handbook on conducting bibliometric analyses by Rehn et al., published in 2014 [25]. The data under analysis were extracted from the Scopus database, a comprehensive and highly regarded platform that indexes a wide array of scholarly literature across multiple disciplines. Known for its extensive coverage, Scopus includes more than 82 million records from over 25,000 peer-reviewed journals, conference proceedings, and patents, encompassing scientific, technical, medical, and social sciences fields. From this reliable source, we gathered data focusing on recent advancements in our understanding of the human microbiome, particularly the oral microbiome and its connections to human health.

The Scopus database was selected due to its extensive multidisciplinary coverage and its capability to perform bibliometric analyses. While other databases, such as PubMed and Web of Science, also provide valuable studies, the decision to use Scopus ensures data consistency and minimizes redundancy in the literature analyzed. This approach aligns with previous bibliometric studies that have also relied on a single database for methodological rigor and analytical depth, such as Zyoud et al. (2022) [26] and Yuan et al. (2021) [27], both of which utilized Scopus to analyze microbiome-related research trends.

**Figure 1:**
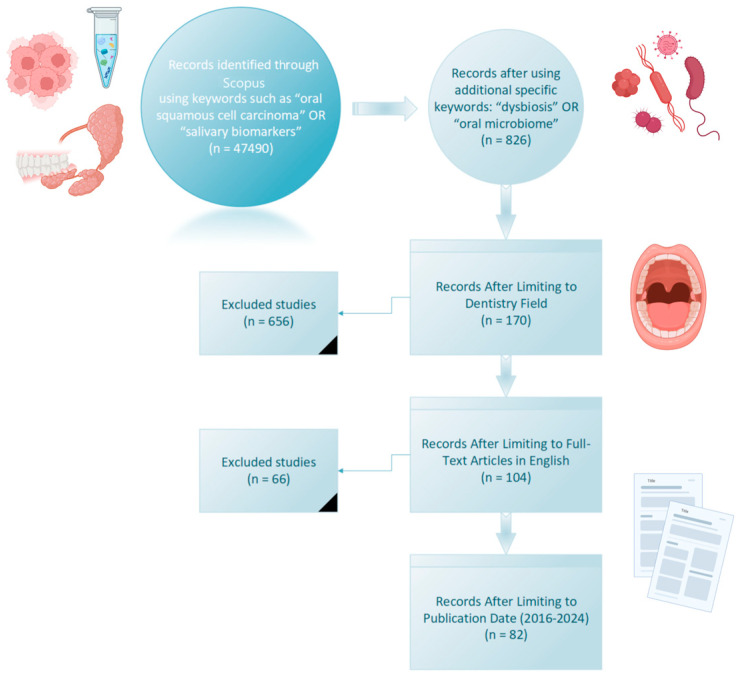
Flow diagram and search strategy in Scopus.

This research, focused on studies conducted on oral cancer and the oral microbiome spanning from January 2016 to April 2024, was carried out in April 2024 to mitigate database renewal bias. The Scopus database was selected for its robust collection of medical literature and comprehensive citation analysis capabilities. The search strategy, depicted in Figure 1, incorporated specific terms related to cancer (e.g., carcinoma, tumor) and the oral region (e.g., oral, mouth), along with terms associated with bacteria and the microbiota, while excluding viral, fungal, and mycotic terms. This meticulous approach ensured the inclusion of relevant and high-quality data pertinent to our study.

Several oral and systemic diseases associated with the oral microbiome were identified, including caries, periodontal diseases, and oral cancer, which have garnered significant attention within the scientific community. A combination of search terms, including “oral squamous cell carcinoma”, “oral microbiome”, “dysbiosis”, and “salivary biomarkers”, was employed using appropriate logical operators to yield relevant results. Initially, a sample of 826 publications spanning from 2016 to 2024 was obtained, which was subsequently narrowed down to 82 documents pertinent to dentistry, excluding other fields and publications not in English.

**Figure 2:**
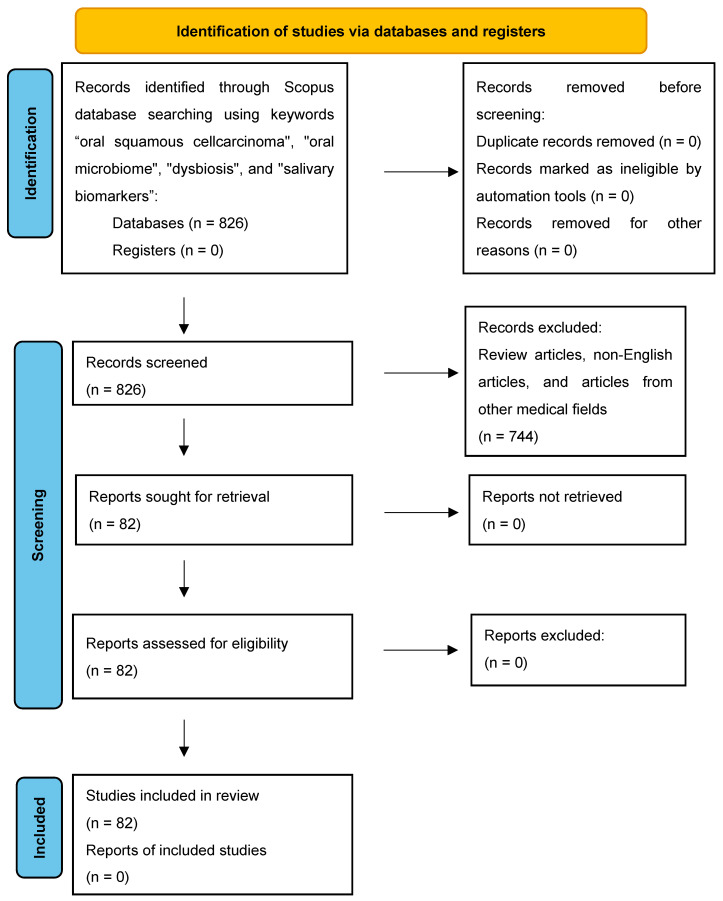
PRISMA 2020 flow diagram for new systematic reviews which include searches of databases and registers only. Registers: organized collections of data, such as databases or repositories (e.g., Scopus), used to systematically identify and retrieve studies relevant to the oral microbiome and oral cancer research included in this analysis.

This bibliometric analysis was conducted in accordance with the PRISMA (Preferred Reporting Items for Systematic Reviews and Meta-Analyses) 2020 guidelines. PRISMA provides a clear set of directives for systematic and meta-analytic reporting, thereby facilitating the evaluation and reproducibility of studies. In this research, we followed the PRISMA-recommended steps, including the formulation of a comprehensive search strategy, rigorous assessment of bias risk, and clear presentation of results via the PRISMA flow diagram. By adhering to these standards, we ensured a robust and transparent methodological approach in exploring changes in the oral microbiome in the context of oral squamous cell carcinoma and identifying relevant salivary biomarkers [28]. This systematic review, conducted retrospectively and focused on bibliometric analysis, adhered to the PRISMA 2020 guidelines to ensure transparency and reproducibility, although it was not pre-registered in a review database (Figure 2) (Supplementary Materials).

### 2.2. Risk of Bias

To systematically evaluate the methodological quality of the included studies, we utilized the ROB2 tool (Cochrane Risk of Bias Tool 2.0). This tool is a widely recognized and validated approach for assessing the risk of bias in randomized controlled trials, focusing on five critical domains: the randomization process, adherence to interventions, completeness of outcome data, measurement of outcomes, and selection of reported results. Each domain was assessed systematically to identify potential sources of bias that could influence the reliability or validity of the study findings. Domain-level evaluations were rated as “Low Risk”, “Some Concerns”, or “High Risk”, with clear criteria guiding each judgment. For example, in the domain of randomization, we assessed whether allocation concealment and sequence generation were appropriately implemented, while for adherence to interventions, we examined deviations from the intended protocol. The assessments were conducted independently by two trained reviewers to minimize subjective interpretation and ensure consistency. In cases of disagreement, discussions were facilitated to achieve consensus, and a third reviewer was consulted to resolve persistent conflicts. This multi-step approach reduced individual bias and strengthened the validity of the evaluation process. To ensure transparency, all judgments were documented with detailed justifications, allowing reproducibility of the evaluation process. Additionally, results were synthesized to provide an overall risk of bias for each study, highlighting areas of methodological strength and weakness. By employing this rigorous and standardized assessment framework, we enhanced the transparency, consistency, and reliability of the included studies’ evaluation, thereby providing a solid foundation for interpreting the findings of this systematic review [29].

### 2.3. Bibliometric Analysis

For data analysis and visualization, we utilized VOSviewer (version 1.6.20), enabling us to identify and explore research trends and clusters of interest in the field of the oral microbiome and associated diseases.

VOSviewer, a freely available software tool, is utilized to construct and visualize bibliometric networks, offering insights into research clusters, current interests, and emerging trends. Such networks encompass scientific publications or journals, keywords, researchers, countries, research organizations, or other specified inclusion criteria terms. These networks are established through various types of links, including citations, scientific co-authorship, co-occurrence, co-citations, and bibliographic connections. As outlined in the VOSviewer Manual, a link signifies a relation or connection between two items, with each link possessing a strength denoted by different positive numerical values corresponding to the type of analyzed items. For instance, the strength of a link in co-authorship networks could represent the number of documents co-authored by two researchers, while in bibliographic coupling networks, it could indicate the number of cited references shared by two documents [30].

Various analyses, including co-authorship, keyword co-occurrence, and co-citation, were utilized to explore interactions and relationships among publications, keywords, and authors in the field of study. These methods deepened our understanding of the evolution and research directions in the oral microbiome and related areas. Descriptive bibliometric analysis captured both objective indexes (e.g., number of publications, authors, affiliations, citation counts, and country/region) and subjective indexes (e.g., keywords). Version 1.6.20 of VOSviewer was employed to generate bibliometric network maps for co-authorship by country and for keywords [30]. To assess research trends and impact in the field of the oral microbiome and OSCC, a Spearman’s correlation analysis was conducted between the number of publications per year and their citation metrics. The analysis revealed a statistically significant positive correlation (ρ = 0.85, *p* < 0.01), indicating a steady increase in research interest and impact. This trend highlights the growing attention toward microbiome-based biomarkers and the integration of artificial intelligence in OSCC diagnostics.

## 3. Results

### 3.1. Study Selection, Data Extraction, and Risk of Bias

The data collection process was conducted by two independent reviewers. Data collection was performed manually using a predefined data extraction form to ensure consistency and transparency. The collected variables included study characteristics, population demographics, interventions, biomarkers analyzed, outcomes, and information on funding sources. Missing or unclear information was managed by excluding studies with incomplete data.

The statistical analysis further supports the observed research growth. The Spearman’s correlation analysis demonstrated a strong positive correlation (ρ = 0.85, *p* < 0.01) between publication counts and citation metrics, confirming an increasing trend in research activity. This suggests that microbiome-based OSCC studies have gained substantial academic and clinical relevance, with a significant rise in interest over the past eight years.

Effect measures, such as mean differences and risk ratios, were used to interpret the results depending on the type of biomarker. Studies were synthesized through tabulation and visual representation, and the methodological heterogeneity observed among the included studies was qualitatively assessed. This heterogeneity was primarily attributed to differences in study designs and biomarker measurement techniques. Reporting bias was not formally evaluated; however, studies excluded due to incomplete data were documented to ensure transparency in the selection process.

The certainty of the evidence was not assessed using formal frameworks, such as GRADE. Nevertheless, the methodological limitations of the included studies were discussed to adjust the overall confidence in the findings. These limitations, including the prevalence of high-risk domains, were considered in the interpretation of results and conclusions.

Both reviewers systematically extracted relevant information from the included studies, using the predefined data extraction form. Any discrepancies or disagreements between the reviewers were resolved through mutual discussion to ensure consistency and accuracy in the collected data. No third-party arbitration was required as all disagreements were resolved collaboratively. No automation tools were used in the data collection process, and all steps were conducted manually to ensure accuracy and consistency.

**Figure 3:**
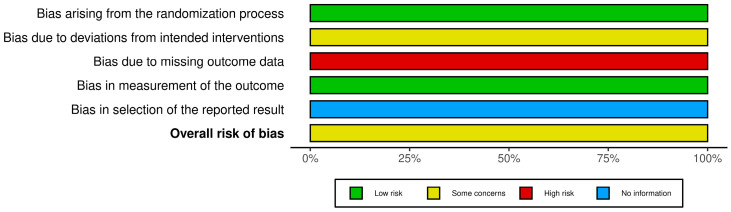
Risk of bias assessment for included studies using the ROB2 tool.

Figure 3 presents the distribution of the risk of bias across the included studies. The most common sources of bias were identified in the domains of “Bias due to missing outcome data” and “Bias in selection of the reported result”, where a significant number of studies were classified as high-risk (red). These findings highlight potential limitations in data completeness and reporting practices, which could impact the overall reliability and validity of the conclusions drawn from these studies. Conversely, the majority of studies demonstrated a low risk of bias (green) in the domains of “Bias arising from the randomization process” and “Bias in measurement of the outcome”, reflecting adequate methodologies in these areas.

### 3.2. Keyword Analysis of Research Themes in Oral Microbiome and Oral Cancer

Between 2016 and 2024, a total of 82 articles on the topic of the oral microbiome, salivary biomarkers, and oral cancer met the search criteria and were included in the evaluation.

These results underscore the need for improved reporting and data management practices in future research. Addressing issues related to missing data and selective reporting could significantly enhance the overall quality of evidence in this field. Moreover, by systematically identifying and visualizing these risks, this assessment provides a foundation for prioritizing methodological improvements in future studies.

**Figure 4:**
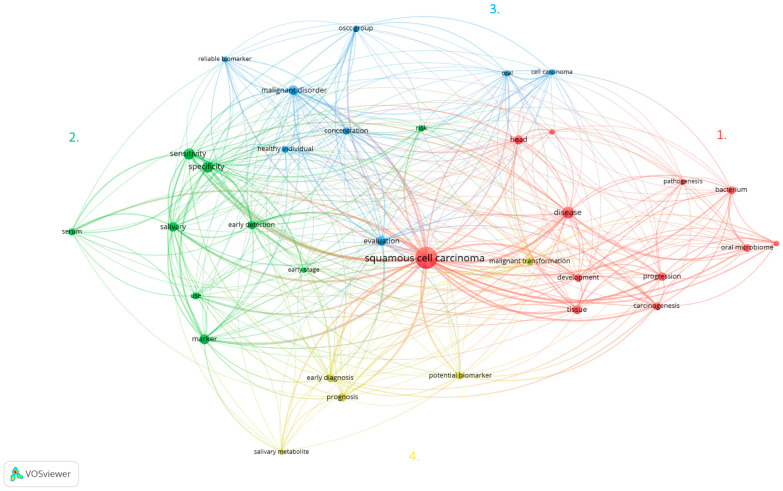
Map of the network of keywords. Source: own processing through VOSviewer.

A keyword analysis was conducted using VOSviewer (Center for Science and Technology Studies, Leiden University, The Netherlands) to discern the predominant research themes in the literature concerning the role of the microbiome in oral cancer development. This method highlights the most relevant keywords based on their frequency, indicated by the number of articles in which a keyword appears at least six times. This means that out of 2408 terms, only 82 meet the threshold, and we chose just the 60% most relevant of them. For our dataset, we chose to generate a map of the network of keywords with a minimum of six occurrences in the analyzed articles, such as oral microbiome (nine occurrences), OSCC (seventy-four occurrences), salivary biomarkers (sixteen occurrences), and early detection (eleven occurrences), in VOSviewer. The most important keywords and the links between them are presented in Figure 4. A larger node (keyword) indicates a greater weight (a higher number of occurrences); a smaller distance between nodes indicates a stronger relationship between them; the same color indicates a series of related keywords or a group of related keywords. Additionally, Table 1 provides a detailed description of these keyword groups, highlighting their occurrences and total link strength within the analyzed dataset.

### 3.3. Analysis of “Co-Authorship” in Terms of Number of Citations

**Figure 5:**
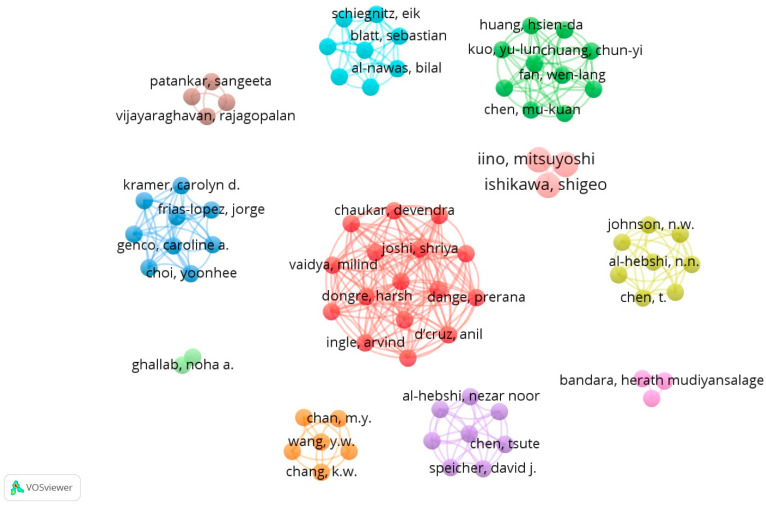
Network of scientific co-authorship, based on the number of documents per author. Source: own processing through VOSviewer.

Research interest in the topic of metabolic pathways can be identified by the number of authors addressing this subject, more concretely through the co-authorship relationships established during scientific research. The analysis in this section focuses on identifying the strongest co-authorship relationships on the analyzed topic, thereby visualizing the most relevant author groups. Out of a total of 553 authors identified in the analyzed articles, 77 of these are cited at least 50 times. Additionally, two authors have met the threshold of at least 50 citations per published document, while four authors have met the threshold of 556 citations per published document. However, their level of collaboration differs, which is why a graphical representation of all 553 authors is not relevant. Individual researchers who do not form clusters with multiple authors are specifically mentioned, while 77 authors have managed to form research teams with significant importance for the analyzed topic. Figure 5 distinguishes the clusters composed of authors collaborating on this topic. Furthermore, the analysis has concentrated on the research domain of the main authors’ network, specifically targeting the primary objective: alterations in the oral microbiome associated with oral and systemic pathology. From the total, 82 articles were selected based on their relevance and citation thresholds to ensure a focused and detailed examination of the collaborative research efforts in this field.

Table 2 organizes the primary authors based on color-coded groups, highlighting the number of citations per document and the total link strength (TLS) for each group. Each cell under the “Main Authors” column lists the authors associated with the respective color group, reflecting their collaborative network and research impact within the domain of oral microbiome alterations related to oral and systemic pathologies. The “Citations/Document” column indicates the minimum citations each author has received per published document, while the “TLS” column shows the cumulative strength of their collaborative links, illustrating the intensity and number of collaborations among these researchers.

### 3.4. Analysis of International Co-Authorship Networks in Scientific Research

To better understand the dynamics of international research collaborations, this bibliometric map provides a detailed visualization of co-authorship networks based on data obtained from Scopus.

This bibliometric map was generated using VOSviewer software and is based on bibliographic data. The analysis was conducted using the “co-authorship” type and the “full counting” method. The parameters of the analysis included a maximum of 25 countries per document, a minimum of one document per country, and a minimum of 50 citations per country. Of the 36 countries that met the citation threshold, 21 were interconnected, resulting in this map.

**Figure 6:**
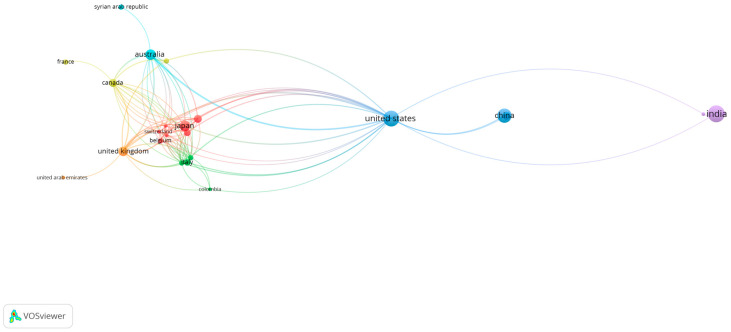
Bibliometric map of international research collaborations.

Figure 6 illustrates the collaboration networks between various countries in scientific publications indexed in Scopus. The United States is shown as a central node with numerous connections to other countries, highlighting its significant role in the global co-authorship network. Other notable countries, such as the United Kingdom, Japan, and Australia, also show multiple international connections. In contrast, India and China, although very active, are depicted with fewer direct connections to other countries, reflecting more limited international collaboration.

### 3.5. Visualizing Collaborative Networks in Dental Research: A Co-Authorship Bibliometric Analysis of Key Organizations

**Figure 7:**
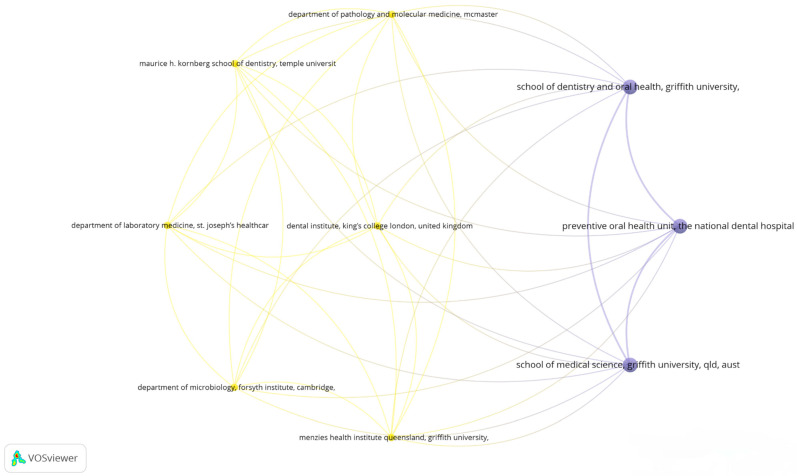
Bibliometric map of collaborations between organizations.

This bibliometric map, created using VOSviewer, visualizes the co-authorship networks among various organizations that have published articles in the field of dentistry, based on data from Scopus. The analysis used the full counting method with a maximum of 25 organizations per document and set thresholds of at least one document and 100 citations per organization. Out of twelve organizations meeting these criteria, nine were interconnected. Key organizations include the School of Dentistry and Oral Health at Griffith University, the Preventive Oral Health Unit at The National Dental Hospital, the School of Medical Science at Griffith University, the Dental Institute at King’s College London, and the Maurice H. Kornberg School of Dentistry at Temple University. The nodes represent organizations, while the links between them indicate the frequency and intensity of their collaborative efforts, highlighting the influential research networks in the field of dental science (Figure 7).

### 3.6. Exploring Co-Occurrence Relationships: Insights from Oral Microbiome Research

In this study, we conducted a detailed analysis of the relationships between key terms in the literature concerning the oral microbiome and associated diseases, using VOSviewer software. For mapping purposes, we utilized bibliographic data downloaded from Scopus and applied various analytical techniques, including co-occurrences, author keywords, and full counting.

After applying a minimum threshold of four co-occurrences, we identified 17 keywords that met this criterion. Among these, “OSCC” and “saliva” were the most frequently encountered, with 38 occurrences. “Biomarkers” were mentioned ten times, while “or cancer” was mentioned twelve times, and the “oral microbiome” was mentioned five times.

**Figure 8:**
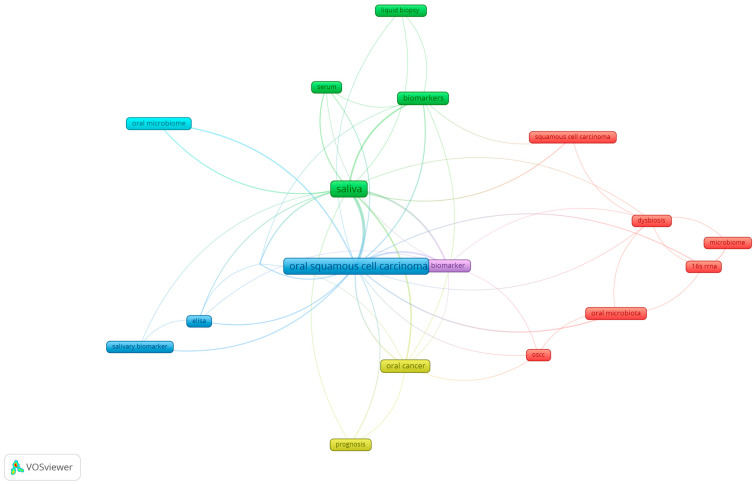
Co-occurrence network analysis of key terms in oral microbiome research.

These statistics provide a clear picture of researchers’ concerns and interests in the field, highlighting the significance of the oral microbiome in the context of oral and systemic diseases. The resulting map enables us to visualize the networks of co-occurrences among these key terms, providing a comprehensive perspective on the interactions within the literature. A detailed analysis of these relationships can contribute to a deeper understanding of the complexity of the oral microbiome and its impact on human health (Figure 8).

## 4. Discussion

Recent insights into the human microbiome have shed light on the intricate role this microbial community plays in our health, especially focusing on the oral microbiome. This deeper understanding has unveiled a wide array of diseases linked to the oral microbiome, encompassing both oral and systemic conditions such as caries, periodontal diseases, oral cancer, colorectal cancer, pancreatic cancer, and irritable bowel syndrome. Moreover, advancements in our comprehension of the human microbiome, with a particular emphasis on the oral microbiome, have highlighted its vital impact on health and disease. This study utilized bibliometric methods to thoroughly review the literature concerning the oral microbiome’s role in oral squamous cell carcinoma (OSCC). The findings showed a notable rise in publications and citations, reflecting the growing interest and acknowledgment of the oral microbiome’s importance in OSCC [17].

The initial focus of oral cancer research primarily centered around epidemiology and the characteristics of oral cancer, likely as part of a population health strategy to identify high-prevalence groups and implement preventive measures. However, there has been a noticeable shift towards investigating cellular/molecular pathways, the tumor microenvironment, the microbiome, therapeutic targets, and biomarkers. These research areas have the potential to empower clinicians to intervene in the pathology at an earlier stage and develop treatment strategies based on specific therapeutic targets, facilitating more precise and minimally invasive treatment approaches. The publication landscape reflects a global interest in oral cancer, with prominent affiliations and countries frequently collaborating on similar research endeavors.

This study offers a thorough assessment of academic publications examining the link between oral cancer and bacteria from 2016 to 2024. The analysis highlights a steady rise in publication numbers over time, indicating a growing interest in this research domain. Furthermore, the citations garnered by these articles exhibit a positive trend, emphasizing their impact and importance in the scientific community. This mirrors the increasing global severity of oral cancer [31] and the pressing necessity to comprehend the role of bacteria in its development [32].

**Figure 9:**
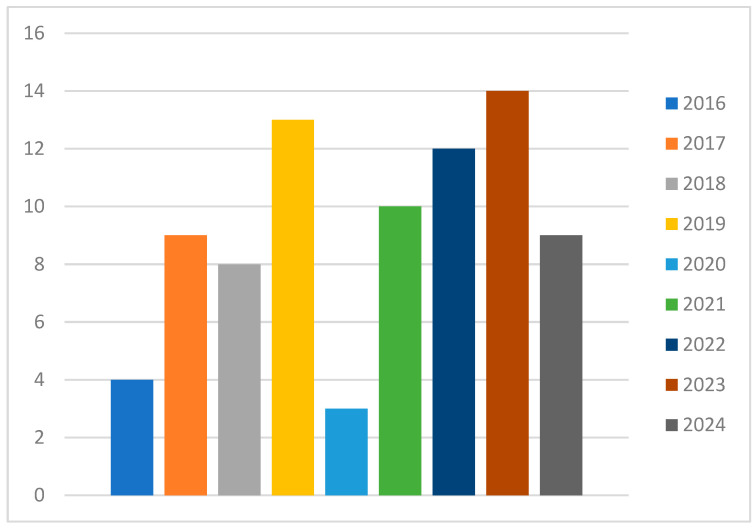
Article appearances across years.

A total of 82 relevant articles on the oral microbiome and oral squamous cell carcinoma (OSCC) from 2016 to 2024 were included, with an average annual publication count of 10.25. The field has shown a steady increase in research interest, reflecting the growing recognition of the oral microbiome’s role in oral cancer. However, during the COVID-19 pandemic, particularly in 2020, there was a noticeable stagnation in publication growth, likely due to research disruptions and the reallocation of resources to pandemic-related studies. Despite the pandemic’s impact, the publication count resumed its growth post 2020, underscoring the field’s resilience and the sustained interest in exploring the oral microbiome’s implications in cancer research. Notably, the 2022 publication count did not reach expected levels, attributed to ongoing pandemic-related challenges and potential publication delays (Figure 9).

Several key studies have contributed to the understanding of the oral microbiome’s role in OSCC. Among the authors investigated, Ahmed et al. in 2024 demonstrated in their study that saliva DNA is a viable medium for detecting somatic mutations in patients with OSCC, with a detection rate of 82% regardless of the tumor stage or primary site, though sensitivity may be limited by low variant allele frequencies (VAFs); while traditional methods such as droplet digital PCR (ddPCR) offer high sensitivity, advancements in panel sequencing and bioinformatics, such as the Integrated Variant Analysis (INVAR) pipeline, may enhance detection capabilities, suggesting a promising avenue for the non-invasive monitoring of OSCC recurrence and treatment response [33]. Rapado-González’s study demonstrates that advanced DNA extraction and analysis techniques, including the use of specialized kits for Formalin-Fixed Paraffin-Embedded (FFPE) tissues and saliva samples, enable the precise identification of genome-wide DNA methylation changes. The methodologies employed, such as bisulfite conversion and methylation panel sequencing, along with sophisticated bioinformatic tools, facilitate detailed DNA methylation profiling, ensuring rigorous quality control and data normalization. These techniques revealed significant differences in DNA methylation and gene expression between OSCC samples and healthy controls, highlighting their potential for disease diagnosis and monitoring [34]. At the same time, Scheurer et al. show the potential of specific miRNA signatures from saliva to distinguish between healthy individuals and OSCC patients, suggesting that standardized protocols for miRNA analysis could be developed to facilitate the early detection of malignant changes, although further validation in larger and more homogeneous studies is necessary to confirm these findings [35]. Zhu et al. indicate that Capnocytophaga gingivalis, highly abundant in OSCC tissues, plays a crucial role in promoting OSCC invasion and metastasis by inducing the epithelial–mesenchymal transition (EMT), suggesting a novel direction for OSCC research focused on the microbial influence on cancer progression [36]. Riccardi et al. in their recent study highlight the potential of salivary transferrin as a biomarker for the early diagnosis of OSCC, emphasizing its role in iron transport and cell proliferation processes; despite promising results indicating suitable sensitivity and specificity, further research is needed to validate these findings and improve early detection strategies for better prognosis in OSCC patients [37,38].

The microbiome profile of OSF-related malignancy showed increased microbial stochastic fluctuation and species co-occurrence network collapse. Artificial intelligence algorithms identified five key species in the OSCC-OSF group, Porphyromonas catoniae, Prevotella multisaccharivorax, Prevotella sp. HMT-300, Mitsuokella sp. HMT-131, and Treponema sp. HMT-927, with robust accuracy in predicting oral carcinogenesis. Functional analysis indicated differences in microbial metabolite potential, suggesting roles in modulating metabolites during oral carcinogenesis. Overall, these findings provide new insights into salivary microbiome alterations during the malignant transformation of OSF [39].

Our analysis unveiled an expanding interest in the oral microbiome within oral cancer research. Studies indicate that dysbiosis in the oral and periodontal microbiome may heighten the risk of oral cancer [40,41].

At the same time, Scheurer et al. highlight in their study the significant role of specific microbial species in oral carcinogenesis, particularly in the transition from normal oral mucosa to OSCC. The genus Gemella, along with species such as Streptococcus, S. agalactiae, and G. haemolysans, were found to be enriched in OSCC samples. These findings suggest that these microbial species may be involved in the metabolic processes associated with cancer development. The study also notes that metabolic pathways, including those related to cysteine and methionine metabolism, are significantly altered in OSCC, pointing to the potential involvement of these microbes in creating a pro-carcinogenic environment. Further research with larger, diverse populations is necessary to confirm these associations and explore their potential as early diagnostic biomarkers for oral cancer [9].

Rani et al. demonstrate the potential of salivary IL-6 as a diagnostic biomarker for oral cancer, particularly OSCC, OPMDs, and CP. Elevated levels of salivary IL-6 were consistently observed in OSCC and moderately in OPMDs, indicating its potential as a reliable indicator of oral malignancy. These findings align with previous research highlighting the diagnostic role of IL-6 in oral cancer, reinforcing its significance as a non-invasive diagnostic tool. Moreover, our study underscores the importance of salivary IL-6 in differentiating between healthy individuals and those with oral lesions, emphasizing its utility in early detection and disease monitoring. While further research is warranted to validate these findings across diverse populations and refine diagnostic criteria, the evidence presented by Rani et al. supports the promising role of salivary IL-6 as a valuable biomarker for oral cancer detection and management [10].

Hashimoto et al.’s study demonstrated significant differences in microbiome profiles in saliva, with an increase in Fusobacteria and Fusobacterium and a decrease in Firmicutes and Streptococcus observed in the OSCC group compared to non-OSCC groups. These findings suggest the potential of these bacterial taxa as novel biomarkers for OSCC detection. Additionally, our analysis of patients with early recurrence suggests the prognostic value of the oral microbiome. Although this study could not elucidate the precise mechanisms by which these bacterial biomarkers influence carcinogenesis and tumor progression, they hold promise for identifying high-risk cases of oral leukoplakia or recurrence in postoperative OSCC patients. Further prospective studies are warranted to validate the clinical utility of oral microbiome profiling in oral diseases [42].

Recent advancements, such as AI-driven models for microbiome analysis, have demonstrated their ability to predict OSCC with high accuracy. For example, Chen et al. (2023) utilized machine learning algorithms to identify microbial patterns associated with early-stage OSCC, achieving a diagnostic accuracy of 92% [43].

By employing keyword analysis with VOSviewer, we identified four main research themes in oral cancer and the microbiome. The first theme emphasized the importance of sensitivity and specificity, focusing on reliable biomarkers like IL-6 and miRNA for the early detection of oral cancer. The second theme explored the interplay between periodontitis, the oral microbiome, and cancer, highlighting how dysbiosis may increase oral cancer risk. The third theme concentrated on malignant disorders and inflammation, examining specific bacteria’s roles in carcinogenesis and chronic inflammation. The final theme addressed evaluation and prognosis, analyzing risk factors and potential markers to develop preventive and diagnostic strategies, ultimately aiming for early intervention and better patient outcomes.

Salivary biomarkers have gained increasing attention for OSCC screening due to their non-invasive nature and potential diagnostic accuracy. For example, Fusobacterium nucleatum, associated with OSCC, can be incorporated into diagnostic panels through targeted qPCR assays, enabling the early detection and monitoring of tumor progression. Similarly, Capnocytophaga gingivalis has shown potential as a microbial marker for distinguishing cancerous from non-cancerous states. These practical applications bridge the gap between theoretical findings and their implementation in clinical diagnostics.

The co-authorship and co-occurrence analyses highlighted significant international and interdisciplinary collaborations. The United States emerged as a central node with numerous connections to other countries, indicating its leading role in global research. Countries like the United Kingdom, Japan, and Australia also showed multiple international collaborations. However, countries such as India and China, despite high research activity, exhibited fewer direct international connections, suggesting a need for enhanced global collaboration.

This study employs bibliometric methods to investigate the progression of research concerning the importance of the oral microbiome and salivary biomarkers in the progression of oral cancer, yielding a comprehensive understanding of various research facets. Nevertheless, it is crucial to recognize and address several limitations in our approach. Firstly, we relied solely on the Scopus database, potentially overlooking relevant articles from other sources. Future studies should consider incorporating multiple databases to ensure broader coverage. Secondly, our focus on original articles might have excluded highly cited review articles, which offer valuable summaries of the literature. Incorporating review articles in future studies could provide a more inclusive overview. Lastly, while citation analysis was utilized, additional quality assessments could enhance the identification of high-quality articles. Despite these limitations, our study furnishes valuable insights into the evolution of research on bacterial influence on oral cancer. Acknowledging these constraints, future studies can build on our findings and employ more robust methodologies. This study contributes to advancing scientific understanding in the field of oral cancer and underscores the imperative for continued research.

This review summarizes findings from multiple studies investigating oral squamous cell carcinoma (OSCC) and related biomarkers, focusing on diagnostic, prognostic, and therapeutic advancements. The studies analyzed in this review demonstrate significant progress in the identification of salivary and serum biomarkers, genetic factors, and microbial changes associated with OSCC. Techniques such as metagenomic sequencing, ELISA, qPCR, and mass spectrometry have been employed to reveal differential biomarker expressions, including microRNAs, cfDNA, cytokines, and metabolites. Statistical analyses like ANOVA, Kruskal–Wallis tests, and ROC curve analysis consistently highlight their diagnostic potential, with several biomarkers achieving high sensitivity and specificity. Moreover, research into the oral microbiome suggests significant microbial diversity alterations in OSCC patients compared to healthy controls. Collectively, these findings emphasize the critical role of molecular and microbial biomarkers in improving early detection, risk stratification, and therapeutic interventions for OSCC (Table 3).

## 5. Conclusions

The bibliometric analysis of the oral microbiome and its association with oral cancer reveals significant growth in research within this field. Our study underscores the increasing recognition of the oral microbiome’s role in the etiology, diagnosis, and prognosis of oral squamous cell carcinoma (OSCC). Notable findings include the identification of numerous salivary biomarkers, such as IL-6, specific miRNA signatures, and microbial profiles, which show promise for the early detection and monitoring of OSCC.

Our analysis highlights key research themes, such as the sensitivity and specificity of biomarkers, the interplay between periodontitis, the oral microbiome, and oral cancer, and the role of specific bacteria in oral carcinogenesis. The importance of international and interdisciplinary collaborations is emphasized by significant co-authorship networks, reflecting strong global interest and cooperation in this research area.

This study advances the scientific understanding of the oral microbiome’s role in oral cancer and emphasizes the necessity of continued research. Future studies can refine the diagnostic criteria and early detection methods, ultimately improving patient outcomes in OSCC.

## Figures and Tables

**Table 1:** Keyword groups. Source: own processing through VOSviewer (Occ., occurrences; T.L.S., total link strength).

Word No.	Group 1 (Red)	Occ	T.L.S.	Group 2 (Green)	Occ	T.L.S.	Group 3 (Blue)	Occ	T.L.S.	Group 4 (Yellow)	Occ	T.L.S.
1	Bacterium	9	36	Early detection	11	59	Cell carcinoma	6	37	Early diagnosis	9	54
2	Carcinogenesis	9	45	Early stage	5	23	Concentration	8	44	Malignant transformation	9	45
3	Development	9	40	Marker	16	70	Evaluation	13	70	Potential biomarker	8	35
4	Disease	20	75	Risk	7	31	Healthy individual	7	38	Prognosis	10	38
5	Head	12	48	Salivary	13	63	Malignant disorder	14	65	Salivary metabolite	5	23
6	Important role	5	25	Sensitivity	19	95	Oral	6	36			
7	Neck squamous cell carcinoma	5	16	Serum	8	34	OSCC group	7	38			
8	Oral microbiome	9	32	Specificity	18	89	Reliable biomarker	5	28			
9	Pathogenesis	5	21	Use	8	35						
10	Progression	10	46									
11	Squamous cell carcinoma	69	277									
12	Tissue	12	43									

**Table 2:** Organization of primary authors by color-coded groups: citations per document and total link strength (TLS). Source: own processing through VOSviewer (T.L.S., total link strength).

Group (Color)	Main Authors	Citations/Document	TLS
Group 1 (red)	Chaukar, Devendra; Joshi, Shriya; Vaidya, Milind; Dongre, Harsh; Iino, Mitsuyoshi	≥50	**75**
Group 2 (light blue)	Schierz, Eik; Blatt, Sebastian; Al-Nawas, Bilal	≥50	**70**
Group 3 (green)	Huang, Hsien-Da; Kuo, Yu-Lun; Tan, Yen-Jang	≥50	85
Group 4 (dark blue)	Kramer, Carolyn D.; Frias-Lopez, Jorge; Genco, Caroline A.; Choi, Yoonhee	≥50	65
Group 5 (yellow)	Johnson, N.W.; Al-Hebshi, N.M.; Chen, T.	≥50	55
Group 6 (purple)	Al-Hebshi, Nezar Noor; Chen, Tsute; Speicher, David J.	≥50	70
Group 7 (orange)	Chang, M.Y.; Wang, W.W.; Chang, K.W.	≥50	55
Group 8 (dark red)	Patankar, Sangeeta; Vijayaraghavan, Rajagopalan	≥50	60
Group 9 (light green)	Ghallab, Noha A.	≥50	50
Group 10 (pink)	Bandara, Herath Mudiyanselage	≥50	45

**Table 3:** Key biomarkers and diagnostic advancements in oral squamous cell carcinoma: a review of current evidence.

Author	Publication Year	Focus of the Investigation	Sample Size	Type of Study	Type of Analysis	Statistics	Results
Lan, Qingying et al. [9]	2023	Microbiota, microflora, Mouth Neoplasms, carcinoma, and squamous cells	18 OSCC patients, 21 OLK patients, and 21 healthy controls (HCs)	Case–control study	The researchers used metagenomic sequencing	Spearman’s correlation	The study found significant differences in the salivary microbiota among OSCC, OLK, and HCs
Daniel, Diana et al. [44]	2021	Biomarkers; oral squamous cell carcinoma; saliva; total sialic acid level	60 subjects divided into three groups	Case–control study	The researchers analyzed total salivary sialic acid (TSA) levels using a sialic acid kit and UV spectrophotometer	Kruskal–Wallis and Mann–Whitney post hoc tests	The study suggested that salivary sialic acid could be a reliable biomarker for detecting OSCC and OPMDs
Yap, T. et al. [45]	2019	Genetics, periodontitis, gingivitis, biomarkers, carcinoma, microRNAs	190 individuals	Systematic review	Developed dysregulation score (dSCORE) and risk classification algorithm	qPCR analysis, dSCORE and risk classification algorithm, sensitivity and specificity analysis, demographic and risk factor analysis	MicroRNA for analysis can be predictably isolated from oral swirls sourced from individuals with a range of demographic, systemic, and oral health findings
Zheng, Jun et al. [46]	2018	Mouth Neoplasms, carcinoma, mouth squamous cell carcinoma, saliva level, early detection of cancer	202 individuals	Observational clinical study	Enzyme-linked immunosorbent assays	Student’s *t*-test, ANOVA, Pearson’s correlation, Spearman’s correlation, ROC analysis	Salivary and serum levels of Naa10p and CEA in OSCC patients were significantly higher than those detected in OPML and the control groups, although patients with OPMLs also showed increased salivary and serum Naa10p and CEA levels as compared to the control group
Hes, Cecilia et al. [47]	2024	Microbiome, Squamous Cell Carcinoma of Head and Neck, metagenomics, DNA library	52 patients	Prospective study	Shotgun metagenomic sequencing	Wilcoxon rank-sum test, Kruskal–Wallis test, Kaplan–Meier survival analysis, Cox proportional hazards model, PERMANOVA, Spearman’s correlation	All patients developed CRT-induced mucositis, including 42% with severe events (i.e., CTCAE v5.0 grade ≥ 3) and 25% who required enteral feeding
Mehdipour, Masoumeh et al. [48]	2023	Biomarkers, squamous cells, microRNA	60 patients divided into four groups	Case–control study	Real-time quantitative polymerase chain reaction (RT-qPCR)	Kruskal–Wallis and Dunn–Bonferroni tests	Altered expressions of microRNA-146a and microRNA-155 in dysplastic OLP and OSCC could serve as potential biomarkers for malignancy
Rapado-González, Óscar et al. [49]	2022	Biomarkers, carcinoma, squamous cells, tumor, Cell-Free Nucleic Acids	34 subjects divided into two groups	Preliminary study	The researchers analyzed the concentration and integrity of cell-free DNA (cfDNA) fragments in saliva samples using molecular techniques	Descriptive Statistics	The study found significant differences in the integrity and quantity of cfDNA between OSCC patients and healthy controls
Menaka, T.R. et al. [50]	2019	Biomarkers; saliva; salivary alkaline phosphatase	42 individuals	Observational cross-sectional study	Kinetic photometric method	Mann–Whitney U test	Data obtained were subjected to statistical analysis; the mean S-ALP was 18.00 IU/L for normal individuals without tobacco usage, 4.60 IU/L for smokers without lesions, 7.50 IU/L for tobacco chewers without any lesions, and 64.90 IU/L for individuals with OPMD
Sridharan, Gokul et al. [51]	2019	Biomarkers, squamous cells, metabolome	61 patients	Observational case–control study	Q-TOF–liquid chromatography–mass spectrometry	MassHunter profile software and Metlin database, ANOVA	Significant upregulation of 1-methylhistidine, inositol 1,3,4-triphosphate, d-glycerate-2-phosphate, 4-nitroquinoline-1-oxide, 2-oxoarginine, norcocaine nitroxide, sphinganine-1-phosphate, and pseudouridine in oral leukoplakia and OSCC was noted
Bandara, Herath Mudiyansalage Herath Nihal et al. [52]	2019	Microbiology, saliva, mouth flora, mycobiome, salivation, biodiversity	20 referenced studies	Systematic review	Literature review, NGS, bioinformatics analysis	Descriptive statistics, ANOVA	Identified Candida as dominant genus; linked fungal diversity to oral diseases like OSCC and periodontitis
Tavakoli, Fatemeh et al. [11]	2024	Mouth squamous cell carcinoma, biological marker, mouth cancer, diagnosis	40 patients with oral squamous cell carcinoma (OSCC)	Observational study	Independent sample *t*-test or its nonparametric equivalent, Mann–Whitney U test	SPSS 17 statistical software	The results showed a statistically significant difference in the mean levels of salivary transferrin between the OSCC patients and the healthy controls
Palaia, Gaspare et al. [53]	2022	miRNAs, OSCC, liquid biopsy	34 studies	Literature search	Qualitative and quantitative review, subgroup analysis	Descriptive statistics, SIGN checklist for bias	The analysis showed that 57 microRNAs of liquid biopsy samples of four different fluids (whole blood, serum, plasma, and saliva) were analyzed
Afifi, Salsabeel et al. [54]	2021	Diagnostic accuracy study, oral cancer, potentially malignant lesions, saliva	28 participants divided into three groups	Prospective pilot study	The researchers used enzyme-linked immunosorbent assay (ELISA)	Chi-square test	Indicated its potential as a promising biomarker for the early detection of oral malignancy
Garg, Ruchika et al. [55]	2017	Oral cancer, oral leukoplakia, oral lichen planus, potentially malignant conditions, survivin	96 subjects	Comparative study	The researchers used high-throughput sequencing and enzyme-linked immunosorbent assay (ELISA)	Mann–Whitney U test	The study found statistically significant differences in the levels of salivary survivin among the groups
Perera, M. et al. [56]	2018	16S, microbiota, ribosomal, RNA, dysbiosis, Sequence Analysis, squamous cells, DNA	The study included 25 cases of oral squamous cell carcinoma (OSCC) and 27 controls with fibroepithelial polyps (FEPs)	Case–control study	The researchers used high-throughput sequencing	Linear discriminant analysis effect size (LEfSe), PICRUSt	The study found that OSCC tissues had lower species richness and diversity compared to FEP tissues
Farshbaf, Alieh et al. [57]	2024	Metabolism, Mouth Neoplasms, biomarkers, squamous cells, biological marker, microRNAs	30 healthy control individuals, 30 patients with erosive/atrophic oral lichen planus (OLP), and 31 patients with oral squamous cell carcinoma (OSCC); this is a cross-sectional study		The researchers used quantitative polymerase chain reaction (qPCR)	Shapiro–Wilk test, Kruskal–Wallis test, Pearson’s chi-square test, Mann–Whitney U test	The study found a statistically significant difference in miR-3928 expression between the three groups (*p* < 0.05)
Nosratzehi, Tahereh et al. [58]	2017	Biomarkers, carcinoma, tumor	75 cases	Observational study	Enzyme-linked immunosorbent assay and optical density	SPSS version 20 and one-way ANOVA and LSD tests were used to analyze the data	The mean salivary endothelin-1 level in patients with OSCC was 163.98 pg/mL; in patients with OLP, it was 160.9 pg/mL; and in healthy people, it was 137.19 pg/ml
Vesty, Anna et al. [59]	2018	Cytokines, head and neck cancer, oral microbiome, oral mycobiome, saliva	30 participants	Observational study	The researchers used 16S rRNA gene sequencing for bacterial analysis and ITS1 amplicon sequencing for fungal analysis	Diversity-based analyses	The study found that the bacterial communities of HNSCC patients were significantly different from those of healthy controls but not from dentally compromised individuals
Blatt, Sebastian et al. [60]	2017	Biomarkers, carcinoma, squamous cells, tumor, cell cycle regulation, Drug Resistance, prognosis	128 studies	Systematic review	Literature analysis, systematic review methodology, narrative synthesis	Not applicable	In the review, the current evidence on over 100 different biomarkers found for predicting prognosis, outcome, and therapy alterations of OSCC is summarized
Saleem, Zohra et al. [61]	2021	Head and Neck Neoplasms, squamous cells, Matrix Metalloproteinase	91 subjects	Analytical study	The researchers used enzyme-linked immunosorbent assay (ELISA)	One-way analysis of variance (ANOVA)	The study found a significant difference in salivary MMP-12 expression between the groups (*p* < 0.001)
Tarrad, Nayroz Abdel Fattah et al. [62]	2023	Biomarkers, Head and Neck Neoplasms, squamous cells, microRNA, precancerous conditions	36 participants	Observational diagnostic study	The researchers used quantitative real-time PCR (qRT-PCR)	Receiver operating characteristic (ROC) curve analysis	The study found that OSCC showed the highest fold change for LINC00657 and the lowest fold change for miRNA-106a among the included groups
Cohen, Erin R. et al. [63]	2020	Saliva, metabolism, biomarkers, progression-free survival, prognosis	64 cases	Prospective study	Immunohistochemistry, salivary biomarker analysis, multivariate analysis	Multivariate analysis, *p*-values for associations	High solCD44 levels associated with strong CD44 tissue expression
Rajaram, Suganya et al. [64]	2017	Biomarkers, carcinoma, N-acetylneuraminic acid, saliva, serum	31 patients	Case–control study	Colorimeter, acidic ninhydrin method	Student’s *t*-test	There is elevated serum and salivary sialic acid level in moderately/poorly differentiated squamous cell carcinoma without any significant change in well-differentiated squamous cell carcinoma
Kitamura, Naoya et al. [65]	2023	Head and Neck Neoplasms, squamous cells, DNA	115 Japanese patients with oral squamous cell carcinoma (OSCC)	Case–control study	The researchers used quantitative real-time polymerase chain reaction (qPCR)	Chi-square test	The study found that 20.9% of the patients were positive for MCPyV DNA
Gaba, Fariah I. et al. [66]	2021	Biomarkers, carcinoma, Head and Neck Neoplasms, sensitivity and specificity, interleukin 8, diagnostic accuracy	17 articles	Meta-analysis	Sensitivity and specificity analysis	Specificities of biomarkers	Specificities of the biomarkers analyzed were found to be IL-8 (0.69; 95%CI 0.66–0.99), IL1-β (0.47; 95%CI 0.46–0.90), DUSP-1 (0.75; 95%CI 0.33–1), and S100P (0.73; 95%CI 0.18–0.99)
Cheng, Y.-S.L. et al. [67]	2017	Metabolism, biomarkers, carcinoma, reverse transcriptase polymerase chain reaction	105 human subjects	Observational study	Bio-Rad CFX96 Real-Time System	Mann–Whitney U test with Bonferroni corrections	Only S100P showed significantly higher levels in patients with OSCC compared to both patients with CPNS (*p* = 0.003) and CPS (*p* = 0.007)
Shabbir, Alveena et al. [68]	2022	Mouth Neoplasms, biomarkers, carcinoma	80 participants	Analytical study	The researchers used enzyme-linked immunosorbent assay (ELISA)	One-way analysis of variance (ANOVA)	The study found that salivary Cathepsin B levels were significantly increased in patients with OSCC compared to healthy controls (*p* < 0.001)
Mougeot, Jean-Luc C. et al. [69]	2019	Streptococcus mutans, bacterial microbiome, cancer radiotherapy, DMFS index, host–bacterium interaction, microbial diversity	31 head and neck cancer (HNC) patients	Longitudinal study	The researchers used high-throughput sequencing of the 16S rRNA gene	Beta-diversity analysis and DMFSs (Decayed, Missing, and Filled Surfaces) scores	The study found significant changes in the oral microbiome at T6 and T18
Ishikawa, Shigeo et al. [70]	2019	Biomarkers, tumor marker, Hyperplasia	48 participants	Case–control study	The researchers used capillary electrophoresis–mass spectrometry (CE-MS)	Multiple logistic regression (MLR)	The study identified six metabolites that were significantly different between OSCC/OED and PSOML
Babiuch, Karolina et al. [71]	2019	Antioxidants, Oxidative Stress, Mouth Neoplasms, biomarkers, squamous cells	60 patients	Prospective study	Salivary biomarkers	Chi-square test; Dunn’s post hoc test; Kruskal–Wallis test; Spearman’s correlation test; Mann–Whitney test	The activity of SOD was significantly higher in the OSCC group in comparison with the OL and control groups
Shan, Jing et al. [72]	2019	Saliva analysis, biomarkers, diagnostic test accuracy study, proteomics, early detection of cancer, interleukin 1 receptor blocking agent	60 saliva samples	Diagnostic test accuracy study	Isobaric tags for relative and absolute quantitation (iTRAQ) method	Enzyme-linked immunosorbent assay (ELISA)	In total, 246 differentially expressed proteins were identified by comparing each two groups, and 21 proteins were differentially expressed when OSCC was compared with both OPMD and control
Katase, Naoki et al. [73]	2023	Squamous Cell Carcinoma of Head and Neck, Cell Line, Western blotting, cell proliferation, Intercellular Signaling Peptides and Proteins, prognosis	60 patients	Case–control study	The researchers used immunohistochemistry (IHC)	Chi-square test and logistic regression analysis	The study found that high expression levels of DKK3 and CKAP4 were significantly associated with advanced stage and poorer prognosis in oral cancer patients
Abdelwhab, Amira et al. [74]	2023	Metabolism, Mouth Neoplasms, biomarkers, Head and Neck Neoplasms, histology, dysplasia	Forty oral potentially malignant disorders	Experimental study	Real-time PCR	Comparison between groups	Mucin1 expression in saliva was significantly elevated in oral potentially malignant disorders when compared with controls
Kallalli, Basavaraj N. et al. [75]	2016	Histopathology, mouth disease, saliva level	60 subjects	Observational study	ERBA-CHEM 5 semi-auto-analyzer	Descriptive statistics and paired *t*-test using the SPSS software	The mean LDH levels were as follows: Group I, 608.28 ± 30.22; Group II, 630.96 ± 39.80; and Group III, 182.21 ± 34.85
Chapple, Iain L C et al. [76]	2018	Periodontitis, gingivitis, peri-implantitis	26 experts	Consensus report	The report involved a comprehensive review	Qualitative analysis and expert consensus rather than quantitative statistical methods	The report introduced a new classification system for periodontal and peri-implant diseases and conditions, emphasizing a more comprehensive approach that includes stages and grades of periodontitis, as well as conditions affecting peri-implant health
Jolivet-Gougeon, Anne et al. [77]	2021	Microbiota, RNA 16S, dental caries, microbial diversity, immunocompromised patient, prevalence	42 papers	Scoping review	Preferred Reporting Items for Systematic Reviews and Meta-analysis Protocols (PRISMA—ScR)	A data-charting form was used	They showed a link between the abundance of Capnocytophaga spp. in the oral microbiota and various local pathologies (higher for gingivitis and halitosis; lower in active smokers, etc.) or systemic diseases (higher for cancer and carcinomas, IgA nephropathy, etc.)
Ishikawa, Shigeo et al. [78]	2020	Saliva analysis, cancer diagnosis, squamous cell carcinoma	60 patients	Case–control study	Salivary biomarker analysis	Multiple logistic regression (MLR); Mann–Whitney U test	Saliva analysis showed significant differentiation between SCC patients and control groups
Banavar, Guruduth et al. [79]	2023	RNA, saliva analysis, biomarkers, cancer staging, genomic RNA, head and neck tumor, sensitivity and specificity	1.175 individuals	Observational study	Machine learning analysis	Receiver operating characteristic (ROC)	The classifier showed a specificity of 94% and sensitivity of 90% for participants with oral squamous cell carcinoma (OSCC) and 84.2% for participants with oropharyngeal squamous cell carcinoma (OPSCC)
Hashimoto, Kengo et al. [42]	2022	Saliva analysis, microbial diversity, Bacteroidetes, high-throughput sequencing, cancer recurrence	86 participants	Observational study	The researchers used next-generation sequencing (NGS)	Fisher’s exact test, chi-square test, logistic regression analysis	The study found significant differences in the abundances of certain bacterial genera and phyla among the groups
Zhong, Xiaohuan et al. [80]	2021	RNA 16S, Mouth Neoplasms, microbial community, community dynamics, oral submucous fibrosis	162 participants	Cross-sectional study	16S rRNA gene sequencing	PERMANOVA; Wilcoxon rank-sum; Kruskal–Wallis	The study found that the oral microbiome of people who chew areca nuts is different from that of people who do not chew areca nuts
Khalil, Marwa et al. [81]	2019	Leukoplakia; oral squamous cell carcinoma; saliva; zinc	45 patients	Randomized clinical trial	Standard spectrophotometric methods	ANOVA and chi-square test	There was a highly significant decrease in the level of salivary Zn in patients with OSCC when compared to OL patients and controls (*p* < 0.05)
Chari, Abinaya et al. [82]	2016	Biomarkers, squamous cells, Surveys and Questionnaires, blood	35 patients	Case–control study	Blood biomarker analysis	Two-tailed *t*-test and chi-square analysis	The mean serum LDH value for patients with smokeless tobacco-related oral lesions was 446.8 U/L, compared to 269.4 U/L for healthy controls
Chen, Jijun et al. [43]	2023	RNA, real-time polymerase chain reaction, biomarkers, Squamous Cell Carcinoma of Head and Neck, cell migration, blood, protein expression	132 participants	Case–control study	Real-time PCR analysis	ANOVA, Tukey test	The study found that miR-19a, GPR39 mRNA, and PKC mRNA were upregulated while GRK6 mRNA was downregulated in the serum and saliva samples of OSCC patients compared to healthy controls
Sayal, Lana et al. [83]	2023	Saliva analysis, biomarkers, Head and Neck Neoplasms, sex difference, early cancer diagnosis, liquid biopsy	133 leukoplakia patients versus 137 healthy volunteers	Observational study	Salivary cf-mtDNA and cfDNA were quantified using Multiplex Quantitative PCR	Chi-square test; Shapiro–Wilk test of normality; nonparametric tests (Mann–Whitney U test and Kruskal–Wallis H test)	The study found that the median scores of cfDNA and cf-mtDNA were significantly higher among HNSCC patients compared to healthy controls and OLK patients
Yuanbo, Zhan et al. [16]	2024	DNA, Papillomavirus Infections, tongue tumor	60 patients	Pilot study	The researchers used 16S rRNA gene sequencing	Wilcoxon test; multiple comparisons using FDR *p*-value correction; Spearman’s rank test	The study found that microbiota diversity was significantly increased in p16-positive patients compared to p16-negative patients
Rani, N. Alice Josephine et al. [10]	2023	ELISA; interleukin 6; oral potentially malignant disorders	60 patients	Cross-sectional comparative study	Saliva samples; ELISA	Shapiro–Wilk test; one-way ANOVA; post hoc Tukey test; Kruskal–Wallis test	The study found that the concentration of IL-6 was significantly higher in the OSCC group compared to the other three groups
Ameena, M. et al. [84]	2019	ELISA; leukoplakia; oral squamous cell carcinoma	90 participants	Comparative study	ELISA test	Statistical analysis was performed using the one-way ANOVA	The study found that salivary TNF-α levels were significantly elevated in patients with OSCC compared to those with leukoplakia and healthy controls
Yost, Susan et al. [85]	2018	Microbiota, squamous cells, transcriptome, Virulence, Metagenome	Small pilot study with a limited number of participants	Pilot study	Metatranscriptome analysis	LEfSe; Kruskal–Wallis (KW) sum-rank test; Wilcoxon test	The study found that Fusobacteria exhibited a statistically significant increase in transcript abundance at tumor sites and tumor-adjacent sites in cancer patients compared to healthy controls
Rapado-González, Óscar et al. [34]	2024	Biomarkers, cancer diagnosis, gene expression, genomic DNA	Study included six consecutive patients	Cross-sectional study	Genome-wide DNA methylation profiling	Principal component analysis (PCA) and ROC curve analysis were assessed to obtain the best models	The study identified a group of novel tumor-specific DNA methylation markers with diagnostic potential in saliva
Feng, Yun et al. [86]	2019	Saliva, metabolism, biomarkers, squamous cells	16 patients	Observational study	The researchers used human protease array kits, enzyme-linked immunosorbent assay (ELISA), Western blot, and immunofluorescence	One-way analysis of variance (ANOVA)	The study found that the salivary protease spectrum was significantly associated with oral diseases
Ghallab, Noha A. et al. [87]	2017	Saliva, metabolism, biomarkers, Intercellular Signaling Peptides and Proteins	45 individuals	Case–control study	The researchers used enzyme-linked immunosorbent assays (ELISAs)	The study employed receiver operator characteristic (ROC) curve analysis	The study found that serum and salivary levels of chemerin and MMP-9 were significantly higher in patients with OSCC compared to those with OPMLs and healthy controls
Sawant, Sharada et al. [88]	2016	Biomarkers, squamous cells, prognosis, CD44 protein, survival rate	87 patients	Prospective study	The researchers performed immunohistochemistry	Chi-square test and Fisher’s exact test	The study found significant correlations between the expression of Oct4, CD44, and c-Myc with overall survival (OS) and disease-free survival (DFS) independently
Lee, L.T. et al. [89]	2018	Mouth tumor, biomarkers, carcinoma, cytokine, blood sampling, interleukin 1 beta, interleukin 6, risk factors	65 patients	Case–control study	Luminex Bead-based Multiplex Assay	Mann–Whitney U test and Kruskal–Wallis test	The study found that plasma levels of IP-10 in early-stage OSCC patients differed significantly from those in controls
Triani, Maulina et al. [90]	2021	Betel nut; Ki-67; micronucleus; oral squamous cell carcinoma; saliva	60 participants	Cross-sectional analytic survey	Papanicolaou method	Mann–Whitney and Kruskal–Wallis tests	The study found significant differences in Ki-67 expression and micronucleus counts between the betel nut chewers with OSCC and the control groups
Perera, Manosha et al. [91]	2017	Mycobiome, carcinoma, DNA ribosomal spacer, microbiome, squamous cells	52 tissue biopsies	Observational study	The researchers used Illumina sequencing and BLASTN algorithm	Linear discriminant analysis effect size (LEfSe)	The study found that the mycobiome in OSCC was dominated by Candida albicans, with significantly lower species richness and diversity compared to FEPs
Chan, Jason Y.K. et al. [92]	2022	Ribosomal, RNA 16S, dysbiosis, head and neck squamous cell carcinoma, genotyping	166 Chinese adults	Cohort study	Permutational multivariate analysis of variance (PERMANOVA)	Mann–Whitney; Wilcoxon rank-sum test	The study found that 15.7% of the HNSCC patients were positive for HPV DNA, with infection rates varying by cancer subtype
Kaur, Jasdeep et al. [93]	2016	Biomarkers, sensitivity and specificity, risk factor, biopsy	200 participants	Observational study	Receiver operating characteristic (ROC) analysis	Mann–Whitney U test	The findings revealed that patients with OSCC and precancerous conditions exhibited significantly higher salivary levels of 8-OHdG and MDA, alongside lower levels of vitamins C and E, compared to healthy controls
Zhu, Weiwen et al. [36]	2024	Squamous cells, Streptococcus, Capnocytophaga gingivalis, Cell Line, Fluorescence, In Situ Hybridization	178 participants	Comparative observational study	16S rRNA gene sequencing	PERMANOVA, *t*-test, ANOVA, regression	The findings revealed that the overall microbiome diversity was higher in healthy controls compared to OSCC patients
Chen, M.Y. et al. [39]	2021	Microbiota, Head and Neck Neoplasms, artificial intelligence	OSF (n = 18) and OSCC-OSF (n = 34) groups	Comparative observational study	16S rRNA gene sequencing	ANOVA, regression, Alpha-diversity indices	The study found significant differences in the salivary microbiomes between the OSCC-OSF and OSF groups
Kumaran, Jimsha et al. [94]	2022	MicroRNA; non-invasive method; potential biomarker	Does not specify the exact number	Observational study	Quantitative real-time PCR (qRT-PCR)	Not applicable	Discusses the differential expression of specific salivary miRNAs and their potential roles as biomarkers for oral diseases
Suresh, H. et al. [95]	2022	Alpha-L-fucosidase; oral squamous cell carcinoma; oral submucous fibrosis; salivary biomarker	40 participants	Comparative study	Enzyme-linked immunosorbent assay (ELISA)	Pearson’s correlation	The study found a significant increase in AFU levels in both saliva and serum of OSMF patients compared to healthy controls
Ferrazzo, Kívia Linhares et al. [96]	2022	Saliva; biomarkers; squamous cells	20 participants	Case–control study	Questionnaire; saliva samples; liquid chromatography–mass spectrometry (LC-MS/MS)	Shapiro–Wilk test; Student’s *t*-test; Mann–Whitney test	The results suggest that while salivary pipecolic acid demonstrates high sensitivity, its specificity is moderate, indicating potential as a non-invasive biomarker for HNSCC detection
SHahinas, J. et al. [97]	2018	Metabolism, biomarkers, carcinoma, squamous cell carcinoma, prognosis, immunohistochemistry	36 articles	Systematic review	Quality in Prognosis Studies (QUIPS), data extraction form	Cox regression and Kaplan–Meier	The findings indicated that the majority of the reviewed studies were replication prognostic factor studies (35 out of 36)
Huang, Long et al. [98]	2022	Biomarkers, carcinoma, diagnostic test accuracy study, diagnostic value	Six studies	Meta-analysis	Hierarchical analysis	Quality Assessment for Studies of Diagnostic Accuracy 2	The pooled sensitivity and specificity of circRNAs for OSCC diagnosis were 0.72 and 0.81, respectively
Furquim, C.P. et al. [99]	2017	Microbiota, saliva, squamous cells, risk factors, Gingival Hemorrhage	61 patients	Cross-sectional	16S rRNA gene sequencing	General linear models	The analysis revealed that the most abundant bacterial phyla in the salivary microbiome of FA patients were Firmicutes and Bacteroidetes
Susha, Karthika Pradeep et al. [100]	2023	Salivary biomarker, oral squamous cell carcinoma, oral leukoplakia, early diagnosis	90 patients	Case–control study	Salivary chemerin; ELISA	Independent *t*-test; analysis of variance test (F test); Scheffe’s multiple comparisons (post hoc test)	The study found that salivary chemerin levels were significantly higher in the OSCC group compared to both the OL and HC groups
Liu, Wei et al. [101]	2023	Bubble analysis; non-invasive diagnosis; oral cancer; oral potentially malignant disorders; saliva; microRNAs	17 eligible studies	Systematic review	Bubble chart analysis	Excel Visual Basic for Applications	The analysis identified that miR-21 exhibited the highest diagnostic power for detecting the onset of OPMDs, followed by miR-31 and miR-142
Peisker, Andre et al. [102]	2017	Biomarkers, carcinoma, biopsy, enzyme-linked immunosorbent assay	60 participants	Case–control study	Saliva samples; enzyme-linked immunosorbent assay (ELISA)	Mann–Whitney U test	The median absorbance MMP-9 value for the OSCC group was 0.186 (IQR = 0.158), while the control group had a median value of 0.156 (IQR = 0.102)
Nakamichi, Eiji et al. [103]	2021	Saliva, biomarkers, carcinoma, Western blotting, immunoreactivity, protein analysis	57 patients	Case–control study	Serum/salivary exoAlix levels	Chi-square tests; Mann–Whitney U tests; Kruskal–Wallis test; Dunn’s tests; Fisher’s exact tests; Spearman’s correlation coefficient by rank; Wilcoxon matched-pairs signed-rank tests	The findings revealed that both serum and salivary exoAlix levels were significantly higher in OSCC patients compared to healthy controls
Nguyen, Truc Thi Hoang et al. [104]	2020	Carcinogenesis, saliva protein, transcriptomics, proteomics, metabolomics, liquid biopsy	This study did not involve a specific sample size	Narrative review	Literature review	Not applicable	The review highlights several salivary biomarkers with potential diagnostic and prognostic value for OSCC
Robledo-Sierra, Jairo et al. [105]	2019	Microbiota, carcinoma, squamous cells, bacterium identification	23 studies on oral squamous cell carcinoma, 2 studies on oral leukoplakia, and 4 studies on oral lichen planus	Systematic review	The researchers performed a comprehensive literature review and meta-analysis	The study employed qualitative synthesis and quantitative analysis	The study found substantial differences in diagnostic criteria, sample types, regions sequenced, and sequencing methods across the included studies
Abdul Aziz Shaikh, Sabiha et al. [106]	2024	TNF-α, oral submucosal fibrosis, squamous cell carcinoma, salivary biomarkers, chronic inflammation	45 participants	Case–control study	Saliva samples; salivary TNF-α levels; ELISA kit	ANOVA and post hoc Tukey HSD	The analysis revealed no significant differences in salivary TNF-α levels among the OSMF, SCC, and control groups
Vimal, Joseph et al. [107]	2023	Biomarkers, Head and Neck Neoplasms, Squamous Cell Carcinoma of Head and Neck	20 samples from patients	Case–control study	Untargeted metabolomics	Progenesis QI	The study identified N-Acetyl-D-glucosamine, L-Pipecolic acid, and L-Carnitine as signature diagnostic biomarkers for oral tongue squamous cell carcinoma
Man, Qi-Wen et al. [108]	2023	Mouth Neoplasms, biomarkers, flow cytometry, protein expression, prognosis	61 patients with OSCC, 21 healthy patients	Short communication	Salivary mRNA	Mann–Whitney test, univariate and multivariate analyses	Identified potential of salivary mRNA biomarkers for OSCC early prediction
Yang, Shun-Fa et al. [109]	2018	Genomics, microbiota, microbial community, histopathology	103 participants	Observational study	16S rRNA amplicon sequencing	Taxonomic analysis	The analysis revealed three distinct MSC groups in OSCC patients, each significantly associated with specific demographic and clinical features
Yang, S.F. et al. [110]	2022	Microbiota, RNA, mouth tumor, Head and Neck Neoplasms, squamous cells	315 participants	Meta-analysis	Sequencing the salivary microbiome	Quantitative trait loci (QTL)	The study identified associations of three genetic loci with the abundance of specific bacterial genera and four loci with measures of β diversity
Tantray, Shoborose et al. [111]	2022	Metabolite, OSCC, OLK	90 participants	Comparative observational study	The researchers employed gas chromatography–mass spectrometry (GC-MS)	Principal component analysis (PCA)	Fifteen metabolites showed significant differences between the control, OLK, and OSCC groups
Gulzar, Rukhsaar Akbar et al. [112]	2021	Salivary MUC1, cancerous conditions	30 patients	Comparative observational study	ELISA	ANOVA	MUC1 levels were highest in OSCC (19.33 ± 4.39 ng/mL), followed by premalignant (8.32 ± 3.08 ng/mL) and control (2.73 ± 0.34 ng/mL)
Gupta, Archana et al. [113]	2019	Mustard oil, dry socket, Curcumin, Holy powder, anti-inflammatory	178 patients	Randomized clinical trial	Comparative analysis of treatment outcomes between two groups	Chi-square test	Group A (turmeric and mustard oil) showed faster symptom relief (starting by day 2) compared to Group B (zinc oxide eugenol, relief by day 4)
Ahmed, Ahmed A. et al. [33]	2024	Saliva, Mouth Neoplasms, DNA sequencing, biomarkers, circulating free DNA, liquid biopsy	250 participants	Observational study	Descriptive and inferential statistics	Nuclear morphometric analysis (NMA) using Image J, receiver operating characteristic (ROC) analysis	Significant differences in nuclear morphometric parameters (NMPs) among different smoking groups
Karmelić, Ivana et al. [114]	2022	Oral cancer; early diagnosis; biomarkers; saliva	58 patients	Cross section	Enzyme-linked immunosorbent assay (ELISA) testing	Mann–Whitney U test and Kolmogorov–Smirnov test	Difference between the OSCC group and the control group was found between the levels of SCCA1 and SCCA 2 both in UWS and SWS
Tang, Kai Dun et al. [115]	2024	Metabolism, biomarkers, Head and Neck Neoplasms, microRNA	66 participants	Pilot study	Quantitative analysis using qPCR (quantitative polymerase chain reaction)	*t*-tests; one-way ANOVA; Tukey–Kramer HSD multiple comparison; Pearson’s correlation	The study identified specific miRNAs (miR-744, miR-150-5P, and miR-146B-5P) with high discriminatory capacity between Fanconi anemia patients and healthy controls

## Data Availability

The data presented in this study are available on request from the corresponding author.

## Supplementary Materials

The following supporting information can be downloaded at: https://www.mdpi.com/article/10.3390/microorganisms13020373/s1. PRISMA 2020 Main Checklist: This document contains the PRISMA 2020 Main Checklist, detailing the methodology used in this systematic review. Relevant sections of this checklist have been incorporated as images within the manuscript for clarity; PRISMA 2020 Abstract Checklist: This document provides the PRISMA 2020 Abstract Checklist, summarizing essential aspects of the systematic review process. Content from this checklist has also been included as images within the manuscript for reference. Reference [116] is cited in the supplementary materials.
